# Supplementary material for: Deletion of wfs1 Impairs Oligodendrocyte Precursor Cells Dorsal Distribution and Myelination Through the wfs1-hmgcs1 Axis in Zebrafish
Source: Biology (Basel). 2026 Aug 16;15(16):1402. doi: 10.3390/biology15161402 (PMC13510151; doi:10.3390/biology15161402)
Supplement: Supplementary file 1 [file biology-15-01402-s001.zip › File S1-Transcriptome sequencing data and differentially expressed genes (DEGs) in wfs1-- zebrafish at 5 dpf/2.KEGG/wfs1KOvsWT/UP/src/dre04744.html]

dre04744


- dre:140425

- Up regulated genes

140425(1.92392776077717)

- dre:140425

- Up regulated genes

140425(1.92392776077717)

- dre:792319

- Up regulated genes

792319(1.01123443865891)

- dre:792319

- Up regulated genes

792319(1.01123443865891)

- dre:792319

- Up regulated genes

792319(1.01123443865891)

- dre:192322

- Up regulated genes

336121(1.42904963424916)

- dre:192322

- Up regulated genes

336121(1.42904963424916)

Close
